# 

**Published:** 1889-08

**Authors:** 


**The** Austin District Medical Society meets in Austin on Friday, 20th of September. A good programme has been arranged. Officers will be elected. The Society will celebrate its second anniversary with a banquet.

---

**The** College of Physicians and Surgeons of Baltimore has its announcement in this issue. Dr. Thos. Opie is Dean. This college is a great favorite with the Texas profession, some of its *Alumni* being among our leading and rising physicians. Write for catalogue.

